# Nano-Encapsulated Antioxidant: Retinoic Acid as a Natural Mucosal Adjuvant for Intranasal Immunization against Chronic Experimental Toxoplasmosis

**DOI:** 10.3390/tropicalmed8020106

**Published:** 2023-02-07

**Authors:** Doaa E. Said, Eglal I. Amer, Eman Sheta, Shaimaa Makled, Fadwa M. Arafa, Hala E. Diab

**Affiliations:** 1 Department of Medical Parasitology, Faculty of Medicine, Alexandria University, Alexandria 5424041, Egypt; 2 Department of Pathology, Faculty of Medicine, Alexandria University, Alexandria 5424041, Egypt; 3 Department of Pharmaceutics, Faculty of Pharmacy, Alexandria University, Alexandria 21521, Egypt

**Keywords:** *Toxoplasma gondii*, retinoic acid, vaccine, mucosal, IgA, adjuvant

## Abstract

The tight relationship between immunity and retinoid levels provides evidence on the critical role of retinoic acid (RA) in regulating immune activity, especially the mucosal one. Mucosal immune response is the key for determination of the outcome of infection, particularly against intracellular mucosal pathogens such as *Toxoplasma gondii*, where it plays a crucial role as a sentinel against parasite invasion. Herein, the immunomodulatory adjuvant role of RA was evaluated for prophylactic vaccination against chronic *Toxoplasma* infection. A quantity of 15 µg of RA pre-encapsulated with lipid-based nanoparticles (SLNs) was intranasally used in three doses, two weeks apart, as an adjuvant to the *Toxoplasma* lysate antigen (TLA). Afterward, mice were infected with 20 cysts of *T. gondii* (ME49 strain) and were sacrificed at the 4th week post-infection. Parasitological, immunological, biochemical, and histopathological studies were applied as vaccine efficacy measures. The protective role of the tested vaccine was noted using the statistically marked reduction in brain cyst count, accompanied by remarkable levels of protective IFN-γ and antibodies, with amelioration of infection-induced oxidative stress and brain pathology. Ultimately, this experiment outlined the prospective role of a novel, natural, nano-encapsulated and mucosal vaccine adjuvant RA-SLNs as a propitious candidate against chronic toxoplasmosis.

## 1. Introduction

Toxoplasmosis is a worldwide, zoonotic protozoal infection with great medical impact. It is caused by the obligate intracellular coccidian protozoan, *Toxoplasma gondii* (*T. gondii*), that is able to infect humans and nearly all homeothermic animals [1]. The host’s genetic background, immune status, and the parasite strain are the major determinants of the immune response against *T. gondii.* In spite of extensive knowledge of *T. gondii* biology, epidemiology, and host–pathogen interactions, there are still few effective control strategies available to prevent humans’ infection and disease [2]. In the current situation, the need for a broadly protective *T. gondii* vaccine is urgent [3]. There are several factors influencing the efficacy of vaccine success including, firstly, vaccine-related features such as immunogen pattern, vaccine type, its components, adjuvant, and regimen; secondly, individual variation among vaccine recipients; and lastly, variable routes of administration [4].

Regarding the vaccine type, various strategies have been investigated to develop a toxoplasmosis vaccine, such as an inactivated and attenuated vaccine, subunit vaccine, recombinant engineered vaccine, and/or DNA vaccine [5]. The immunogenicity of live-attenuated vaccines can be substantially influenced by the degrees of attenuation. Additionally, the use of these vaccines has been restricted due to a lack of reasonable shelf-life, together with the possibility of reverting to a pathogenic strain. Recombinant proteins and/or DNA vaccine induced partial, stage-specific immunoprotection against toxoplasmosis [6]. Meanwhile, a wide range of protective antigens, the majority of which are peptides, lipids, and sugars are incorporated in *Toxoplasma* lysate antigen (TLA) [7]. It is confirmed to have enough antigenicity for murine immune system that is capable of activating all types of immune responses [6,8].

A limited number of studies have addressed the role of chosen route of administration on the vaccine potency. The inoculation route can influence vaccine outreach, which can control the primed immune cells to elicit immune responses both locally and systemically [9]. In low-income developing communities, parenteral vaccines have downsides such as unsafe practices, lack of compliance, and high cost of mass immunization. In addition, they rarely induce detectable mucosal immunity. Eliciting protective immunity both mucosally and systemically through selecting mucosal routes continues to be a difficult task [10]. Mucosal vaccines provide front-line protection against pathogen invasion and dissemination that also mimic the natural pathway of pathogen transmission. The majority of mucosal vaccines are administered orally or nasally. As the nasal cavity has less proteolytic activity, the intranasal route requires fewer antigens than the oral one. Moreover, the highly vascularized nasopharyngeal mucosal surface enhances vaccine absorption and induces immune protection at nasal, interconnected oral, and distant mucosal places such as those rectally and vaginally situated [11].

Several studies have illustrated that the use of appropriate immune modulating compounds can have a positive impact on vaccine efficacy. Hence, immune modulators and adjuvants are critical components of modern vaccines, especially those promoting antibody response on mucosal surfaces. The inclusion of efficient and safe mucosal adjuvants in vaccine formulation remains a priority in order to enhance both mucosal and systemic immune responses [12]. The most commonly described mucosal adjuvants include emulsion-based adjuvants, synthetic and natural microbial derivatives, and cytokines. Unfortunately, the majority of them were never accepted for routine vaccination because of safety concerns such as acute toxicity and the possibility of delayed side effects [13]. As a result, the development of new and improved mucosal vaccine adjuvants is critical.

As safety has been the major issue in development of a novel vaccine adjuvant, naturally occurring antioxidants with immune-modulatory action have been studied for their possible use as vaccine adjuvants. Moreover, oxidative stress (OS) plays a critical role in the development of *Toxoplasma* encephalitis (TE) neuropathology [14]. Hence, the scientific community is currently looking for effective, naturally occurring antioxidants to be used effectively for immune system modulation [15].

Retinoic acid (RA), a vitamin A-active metabolite, presents naturally in several animal and plant sources. It is crucial for the conservation and mediation of various physiological and biological processes including augmentation of the immune response, and epithelial and mucosal tissue homeostasis [16]. Additionally, it has astrong scavenging activity against a spectrum of reactive oxygen species (ROS). However, RA clinical applications have revealed three major limitations. Firstly, their photosensitivity and poor solubility in aqueous solutions [17]. Secondly, RA induces mucocutaneous irritation on topical application and increased catabolism when it is administered intravenously. Lastly, the systemic delivery of RA causes side effects including headache and hypertriglyceridemia, especially when administered at high doses. All these limitations have motivated researchers to develop novel retinoid delivery formulations, which have been modified in some cases to target specific tissues and cells of interest [18].

Solid lipid nanoparticles (SLNs) have been explored as an alternative carrier for therapeutic peptides, proteins, antigens, and bioactive molecules. Similar to emulsions and liposomes, SLNs consist of physiologically well-tolerated elements, and provide the opportunity of controlled adjuvant release. Moreover, in a similar way to polymer-based nanoparticles, the solid matrix protects active ingredients from chemical degradation, which offers remarkable improvement in adjuvant release characterization. SLNs can be manufactured without toxic solvents, and they can be produced widely [19]. Moreover, the lipids used as the matrix of SLN are well-tolerated and have a generally regarded as safe (GRAS) rating and low toxicity [20].

Consequently, this experimental study was carried out to investigate the impact of a naturally occurring antioxidant—RA loaded SLNs—as new mucosal adjuvant in enhancing the potency of intranasal immunization by using TLA against chronic experimental toxoplasmosis. 

## 2. Materials and Methods

### 2.1. Ethics Statement

Based on Egyptian regulations for animal experimentation, the Ethics Committee of the Alexandria Faculty of Medicine approved all experiments involving animal housing and sacrifice (protocol approval number: 0201455).

### 2.2. Parasites

The current study employed two strains of *T. gondii*. They were kindly provided by the Medical Parasitology Department of the Faculty of Medicine in Alexandria. Antigen preparation and tachyzoite invasion and replication assays were performed using the virulent RH strain. For the challenging infection, the avirulent ME49 strain was used.

### 2.3. Preparation of Antigens

The virulent *T. gondii* RH strain tachyzoites were harvested from infected mice’s peritoneal fluid and processed for antigen preparation [8]. The Bradford method and the Bio-Rad protein assay reagent were used for determination of protein concentration [21]. The prepared Ag (TLA) was stored at −20 °C after being aliquoted until further usage.

### 2.4. Formulation of Plain and RA-SLNs

A modified hot melt homogenization method was used for the preparation of plain SLNs, oily (OP) and aqueous (AP) phases were weighed separately and heated to 85 °C. The OP comprised compritol (1 *w*/*v*%), stearylamine (0.01 *w*/*v*%), and cholesterol (0.05 *w*/*v*%), while 4% PVA (60% of formulation volume) was used as the internal AP. Maintaining the temperature at 85 °C, AP was gently dropped into the melted OP with constant homogenization at 20,000 rpm for 10 min. The formed dispersion was introduced to cold water dropwise (external aqueous phase represents 40% of formulation volume), which was maintained in an ice bath and was homogenized at 10,000 rpm for 10 min. The SLNs’ pH was adjusted to 7.5 with a solution of 0.01 M HCl. For RA-SLN preparation, the same procedure was carried out with the addition of RA (0.01 *w*/*v*%) to the oily phase [22,23]. Lyophilization (VirTis^®^ sentry2.0 lyophilizer (SP Industries, Hopkins, MI, USA)) was conducted for determination of SLN concentration and adjuvant loading.

### 2.5. Nanoparticles’ Profiles

#### 2.5.1. Physical Characterization

The particle size, polydispersity index (PDI), and zeta potential (ZP) of plain SLNs and RA-loaded SLNs were measured using a NanoZS/ZEN3600 Zetasizer (Malvern Instruments Ltd., Malvern, UK) [24].The mean values were calculated after three measurements.

#### 2.5.2. Transmission Electron Microscopy (TEM)

TEM imaging of the newly synthesized particles was performed using a Joel JEM-1400 plus transmission electron microscope (Tokyo, Japan) with an accelerating voltage of 80 kV [24].

#### 2.5.3. Determination of Entrapment Efficiency (EE%)

EE% was calculated indirectly using a modified centrifugal ultrafiltration technique and a Centrisart^®^-I tube (MWCO 300 kDa, Sartorius AG, Goettingen, Germany). In brief, 1 mL of RA-SLNs were loaded in the filter device and centrifuged at 10,000 rpm, 4 °C, for 12 min. The filtrate was collected and the adjuvant content was quantified using a UV spectrophotometer, (Cary 60 UV-VIS, Agilent technologies, Kyoto, Japan) after determination of the appropriate wavelength for the adjuvant (340 nm) [24]. 

The *EE* was calculated as follows:

$$EE\;\left(\%\right)=\left[\frac{\mathrm{Entrapped}\;\mathrm{adjuvant}\;\mathrm{concentration}\;\mathrm{in}\;\mathrm{the}\;\mathrm{SLNs}\;\left(\mathrm{mg}/\mathrm{mL}\right)}{\mathrm{Initial}\;\mathrm{adjuvant}\;\mathrm{concentration}\;\left(\mathrm{mg}/\mathrm{mL}\right)}\right]\times 100$$

The amount of adjuvant in a specific weight of the freeze-dried SLNs was also calculated to estimate the adjuvant loading (*AL*).

$$AL\;\left(\%\right)=\left[\frac{\mathrm{Total}\;\mathrm{amount}\;\mathrm{of}\;\mathrm{adjuvant}\;\mathrm{in}\;\mathrm{the}\;\mathrm{SLNs}\;\left(\mathrm{mg}\right)}{\mathrm{Total}\;\mathrm{amount}\;\mathrm{of}\;\mathrm{SLNs}\;\left(\mathrm{mg}\right)}\right]\times 100$$

#### 2.5.4. Release Study and Modeling of the Release Kinetics

This was conducted in triplicates using dialysis bag technique. One mL of RA-SLN (0.01% RA) dispersion was placed in dialysis bags immersed in a 10 mL release medium (deionized water and ethanol (50:50, %*v*/*v*)) in firmly closed conical flasks. The flasks were agitated in a temperature-controlled water bath at 37 ± 0.2 °C, 100 rpm. At zero, 0.5, 1, 2, 4, 6, 24, 48 hrs, 5 mL of the release medium were removed and substituted with an equivalent volume of new medium to keep the sink condition. Samples were assayed for RA concentration spectrophotometrically at 340 nm [25]. The release data obtained from dialysis bags were fitted to various release kinetic models (zero-order, first-order, Higuchi, Hixson–Crowell, Korsmeyer–Peppas).

#### 2.5.5. Stability Study

The stability of the dispersions of the RA-SLNs, kept at 4 °C, was assessed at 1, 2, 3, and 6 months, in comparison to the freshly prepared RA-SLNs (at 0 time). Samples were rechecked for their physiochemical characters (particle size, PDI, ZP, and EE%) [26].

### 2.6. Experimental Setup and Immunization Protocols

Sixty male Swiss albino strain mice weighing 20–25 g and aged four to six weeks were used. They were divided into five groups (twelve mice each):

Group I (control group): each mouse was inoculated with sterile PBS. Group II: RA-SLN-inoculated mice, each was administered 15 µg/dose [27]. Group III: TLA-inoculated mice; each was administered 20 µg/dose [8]. Group IV: TLA/plain SLN-inoculated mice; each was administered 20 µg/dose and 130 µg/dose of TLA and plain SLNs, respectively [28]. Group V: TLA/RA-SLN-inoculated mice; each was administered a suspension containing 20 µg/dose and 15 µg/dose of TLA in PBS and RA-SLNs, respectively. Mice from all groups were intranasally (IN) inoculated after being anaesthetized intraperitoneally with 0.3 mL of ketamine (10 mg/mL) and xylazine (1.0 mg/mL) diluted in 0.9% NaCl. The mice remained on their backs until they recovered completely from anesthesia. Two booster doses with two week intervals were administered [8]. Two weeks after the last booster dose, blood samples were drawn from six mice in each group after being anesthetized into Eppendorf aliquots for future studies. Following that, mice were cervically dislocated, and then the intestinal segments and spleens were removed for further immunological research. Brains were also harvested for biochemical studies.

Two weeks after the second booster dose, the rest of the mice were infected orally with 100 μL of the brain homogenate containing 20 cyst of ME49 *Toxoplasma* strain (non-lethal dose) [29]. At the 4th week, following challenging infection, mice from each group were anaesthetized and sacrificed as previously stated. Serum, intestinal segments, brains, and spleens were removed for different assessed parameters.

### 2.7. Vaccine Evaluation

#### 2.7.1. Parasitological Studies

In vitro tachyzoite invasion and replication assay: 

Following the protocol described in Wagner et al., 2015, this assay was conducted to evaluate the capability of serum antibodies to inhibit tachyzoite invasion and replication in cultured Vero cells [7]. Sera of the collected blood samples were aliquoted and kept at −20 °C after filtration using Millex Syringe Filters (0.22 µm). At a concentration of 2.5%, the sterile sera were diluted with complete culture media (RPMI1640 with L-Glutamine; 4% FCS, 1% penicillin/streptomycin). Tachyzoites (5 × 10^5^) were then mixed with the sterile diluted sera. Vero cells were seeded in 48-well plates (4 × 10^4^ cells in 500 µL medium per well) and suspended in complete culture media. Tachyzoites were then introduced into the cultured Vero cells. The medium containing the free tachyzoites was removed after 5 days of cultivation under standard conditions (5% CO_2_, 37 °C), and the total number of tachyzoites/well was determined. Then, for live staining, 10 µL of tachyzoites per well were counted in aliquots using trypan blue. The test was carried out in triplicate.

Determination of brain cyst count and size: 

The total brain cyst burden was determined on day 70 of the experiment. The following equation was used to calculate the percentage of reduction (%R):

%R = (C − W/C) × 100

C: Total cyst count in the control group of mice.

W: Total cyst count in each group of mice.

Using a calibrated ocular micrometer, the harvested cysts size of each group was calculated under the high objective lens (40×) [30].

Scanning electron microscopy (SEM) for cyst ultrastructural changes:

Brain cysts were harvested from three different studied groups (I, III, V), and examined using SEM, after preservation in 2.5% glutaraldehyde solution [30].

#### 2.7.2. Immunological Study

As previously stated, samples of mice sera, intestinal segments, and spleens harvested at day 42 and day 70 of the experiment were collected from all groups and used for immunological assay.

Total IgG levels in serum:

According to the manufacturer’s instructions, mouse *Toxoplasma* IgG ELISA Kit (Chongqing Biospes Co., Ltd., Chongqing, China) was used for measurement of total IgG antibody response. Optical densities (OD) were measured at 450 nm using a microplate reader (TECAN) [8]. 

Gut lavage and secretory IgA levels in intestinal wash:

Each mouse’s intestine was meticulously excised. The mice were fasted for 8 hrs prior to sample collection to deplete the intestinal contents [8]. One ml of cold PBS (pH 7.2) supplemented with 1% (*v*/*v*) anti-protease cocktail (PMSF, SIGMA) was used to wash out the intestinal segments. Mucosal washes were then vortexed and centrifuged (2000 rpm for 30 min at 4 °C). Samples were aliquoted and stored at −20 °C for subsequent S-IgA quantification using mouse *Toxoplasma* antibody IgA ELISA Kit (Chongqing Biospes Co., Ltd.). At 450 nm, optical densities (OD) were measured using a microplate reader (TECAN).

Interferon gamma (IFN-γ) concentration:

The spleens of sacrificed mice from all groups were collected. Each spleen was minced in a petri dish containing 2 mL of RPMI 1640 medium and separated by sieving through a 40-μm cell strainer. The cell suspensions were added to a centrifuge tube and centrifuged at 2000 rpm at 4 °C for 5 min. The supernatant was decanted, and the red blood cells were lysed using red cell lysis buffer. The pellet was washed four times using RPMI medium after centrifugation. Then, using trypan blue, live cells were counted in 10 µL of the suspension. The cells were resuspended in complete RPMI 1640 medium. The cell concentration was adjusted to 2 × 10^6^ cells/mL. Suspensions, 100 µL each, were tittered into 96-wells of a flat-bottomed tissue culture plate and stimulated with TLA at a concentration of 20 μg/mL. The cultures were incubated at 37 °C and 5% CO_2_ for 72 h. Supernatants from cultures were collected using centrifugation at 2000 rpm for 10 min and stored at −20 °C until cytokine quantification. Cytokine (IFN-γ) levels were measured in the cell cultures supernatants using mouse IFN-γ ELISA kit (Chongqing Biospes Co., Ltd.). OD was measured spectrophotometrically at 450 nm using an ELISA reader [8].

#### 2.7.3. Biochemical Study

One hundred mg of the brains from all groups were collected on days 42 and day 70 and processed for determination of oxidative stress biomarkers and the brain samples were perfused with a PBS solution, (pH 7.4.) containing 0.16 mg/mL heparin. The brain tissue was then homogenized in one ml of cold buffer. The homogenate was centrifuged for 15 min at 4 °C at 4000 rpm. The supernatant was collected and kept at −20 °C until needed. 

Superoxide dismutase (SOD):

The superoxide dismutase activity in the brain homogenate supernatants was measured according to manufacturer’s instructions (Biodiagnostics, Giza, Egypt) using the method of Sun et al., 1988. A microplate reader was used to measure absorbance at 560 nm. The results were expressed in units of U/g tissue [31]. 

Malondialdehyde (MDA) (lipid peroxide) assay:

According to Draper and Hadley, 1990, the amount of MDA was determined using the colorimetric lipid peroxidation assay kit (Biodiagnostics, Giza, Egypt) as directed by the manufacturer. At 534 nm, the absorbance was measured spectrophotometrically. MDA concentrations were measured in nmol/mg of total protein [32].

Total Antioxidant Capacity assay (TAC):

The Total Antioxidant Capacity (TAC) colorimetric assay kit was used to measure the total antioxidant concentration in serum samples. The absorbance was measured using microplate reader at 505 (500–510 nm). The results of TAC levels were expressed in mM/L [33].

#### 2.7.4. Histopathological Analysis

On day 70 of the experiment, a portion of the brain tissues from all studied groups were fixed in 10 % formalin solution and assessed for any pathological findings using hematoxylin and eosin (H&E) staining. Grocott–Gomori methenamine silver stain (GMS) was used to stain the cyst wall and bradyzoites [34,35].

### 2.8. Statistics

The Statistical Package for Social Sciences software (SPSS), version 20.0, was utilized. The *F*-test (ANOVA) followed by the post-hoc test (Tukey’s test) was employed for data analysis. A *p*-value less than 0.05 was considered statistically significant. 

## 3. Results

### 3.1. Plain and Adjuvant-Loaded SLN Characterization

#### 3.1.1. Physical Characterization

**Figure 1:**
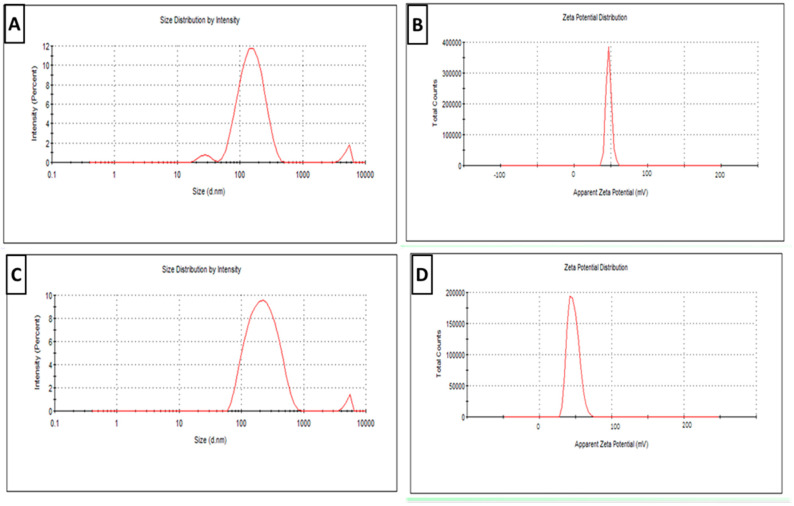
Physical characterization of newly prepared plain and RA-SLN dispersions (zero day). (**A**) The particle size distribution curve for plain SLNs. (**B**) The plain SLNs’ ZP curve. (**C**) The RA-SLN particle size distribution curve. (**D**) The RA-SLNs’ ZP curve.

Plain SLN particle size distribution curves revealed a mean particles size of 165 ± 3 nm and PDI of 0.18 ± 0.05 (<0.5) (Figure 1A). Plain SLNs had a ZP of 48 ± 0.5 mV (Figure 1B). However, the particle size of newly prepared RA- SLN dispersion (at zero day) was 326 ± 6 nm, the PDI was 0.27 ± 0.3 (Figure 1C) (<0.5 indicating a narrow particle size distribution), while the ZP was 47 ± 0.6 mV (Figure 1D).

#### 3.1.2. TEM

**Figure 2:**
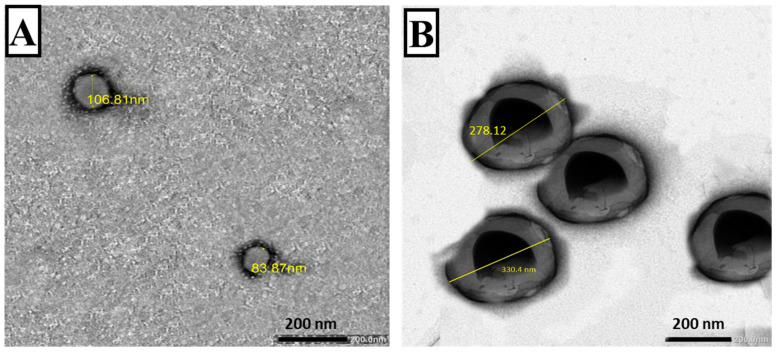
TEM of the prepared nanoparticles (plain and RA-loaded SLNs) (×30,000). (**A**) Plain SLNs displaying round smooth surfaces. (**B**) RA-SLNs showing almost round particles with darker core.

The transmission electron micrographs of plain and RA-SLNs showed that plain SLNs were sphere-shaped with no claustration nor agglomeration (Figure 2A), while the TEM micrograph of RA-SLNs revealed regular spherical particles with darker inner core (Figure 2B).

#### 3.1.3. Entrapment Efficiency and Adjuvant Loading

The EE% and AL% values of the RA-SLNs were found to be 89.1 ± 0.6% and 1 ± 0.1%, respectively.

#### 3.1.4. Release Study and Modeling of the Release Kinetics

**Figure 3:**
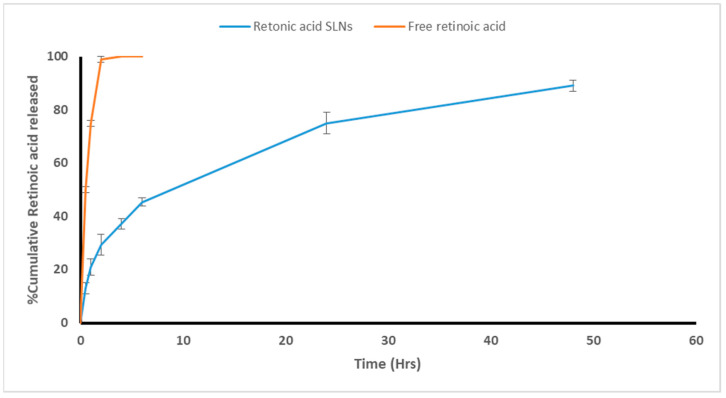
In vitro release profile of RA.

To ensure RA solubility, an in vitro adjuvant release study was performed using the dialysis bag method in 50% ethanol at 37 °C. Additionally, to obtain release profiles, the cumulative amount of the adjuvant released was plotted versus time (Figure 3). Within the first two hours, there was instant diffusion and release of free RA (99%). Adjuvant release from the SLNs, on the other hand, exhibited a biphasic release pattern with an immediate release followed by a sustained release rate. Within the first two hours, 29% of the RA was released (a burst release), followed by sustained adjuvant release from the lipid matrix and core (a slow and controlled release) with 89% of the RA being released from the SLNs’ dispersion within 48 h (Figure 3). The cumulative amount released from adjuvant-loaded SLNs, and its corresponding free form differed significantly (*p* ≤ 0.05). Fitting the release data to different release kinetics models revealed that it is mostly correlated to the Korsmeyer–Peppas model with an R^2^ value of 0.9975 (n = 0.332), which suggests that a Fickian diffusion is prevalent.

#### 3.1.5. The RA-Loaded SLNs’ Stability

RA-loaded SLN dispersions were stored in airtight glass vials at 4 °C. During the storage period, no obvious precipitation or agglomeration was observed. They retained their colloidal characteristics, where there was no significant change in their physicochemical characters (particle size, PDI, zeta potential, and EE) (*p* > 0.05) (Table 1). Across a six-month storage period at 4 °C, RA-loaded SLN dispersions maintained physicochemical stability and EE% with no adjuvant expulsions.

### 3.2. Evaluation of the Vaccine

#### 3.2.1. Parasitological Studies

**Figure 4:**
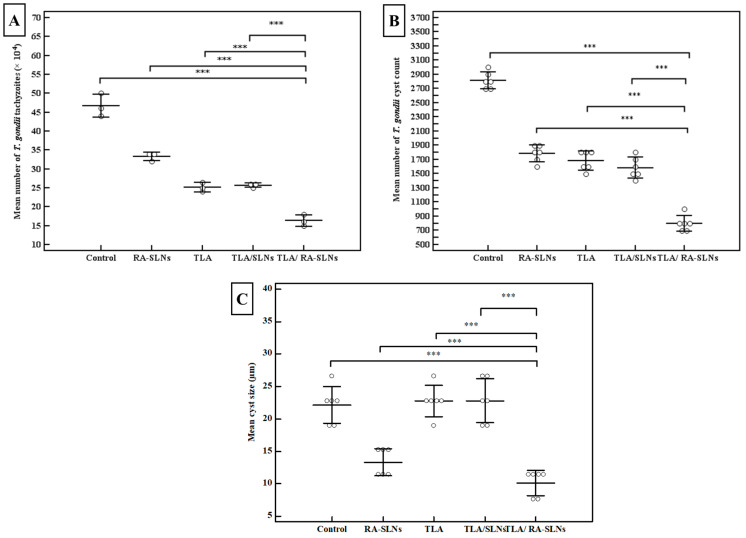
Results of the parasitological studies. (**A**) The tachyzoite invasion and replication assay. The tachyzoites were pre-incubated with sera collected on day zero from naive mice, or sera collected on day 42 from the start of the experiment (before infection) from mice vaccinated with various vaccination protocols. The dots symbolize the mean of *T. gondii* tachyzoites/well (×10^4^) in the supernatant of Vero cell culture after incubation. All in vitro assays were performed using pooled serum samples in triplicates and the data shown represent the results of three independent experiments, (**B**) the mean of *T. gondii* cyst count and (**C**) cyst size in brain of mice of different evaluated groups at the 4th week after the in vivo infection. Data are expressed as mean ± SD. *** *p* < 0.001.

In vitro tachyzoite invasion and replication assay:

When tachyzoites were incubated with sera from TLA-vaccinated mice, there was a significant reduction in tachyzoite count compared to the control PBS group. Still, the mean number of tachyzoites in the culture supernatant was not significantly changed either in the TLA group or TLA/plain SLN group (*p* > 0.05). Meanwhile, encapsulation of RA into SLNs showed enhancement of TLA efficacy, where sera from mice intranasally vaccinated with TLA/RA-SLNs showed the most dramatic reduction in tachyzoite count among all studied groups with a mean number of tachyzoites of 16.33 ± 1.53 × 10^4^ per well, which was statistically significant in comparison to the control PBS group, RA-SLN group, TLA group and TLA/plain-SLN group (*p* < 0.001) (Figure 4A). This regimen resulted in the highest inhibition of tachyzoite invasion, with only 35% of tachyzoites successfully invaded the Vero cells (Table S1, Supplementary Materials).

Brain cyst count and cyst size:

Concerning cyst count in the brain homogenate, the mean *T. gondii* cyst count in the infected control group was 2816.67 ± 117 cysts. Although TLA vaccination, either alone or combined with plain SLNs, significantly reduced the brain cyst load when compared to the infected control group, with a percentage reduction of 40.2 and 43.8, respectively, there was no significant difference between the TLA group and TLA/plain SLN group. Meanwhile, intranasal inoculation of TLA combined with RA-SLNs significantly enhanced TLA immunogenicity together with a dramatic decrease in brain cyst count with the highest recorded percentage of reduction of 71.6% among different studied groups (Figure 4B). Furthermore, they demonstrated significantly less brain cyst load compared to mice preimmunized with TLA either alone or combined with plain SLNs.

Regarding the cyst size, the control group had a mean cyst size of 22.17 ± 2.86 µm, while mice-vaccinated with RA-SLNs either alone or combined with TLA exhibited a statistically significant reduction in cyst size among all studied groups with a mean of 10.13 ± 1.96 µm (Figure 4C). Under the same circumstances, mice vaccinated with other regimens resulted in a non-significant reduction in cyst size when compared to the control group.

**Figure 5:**
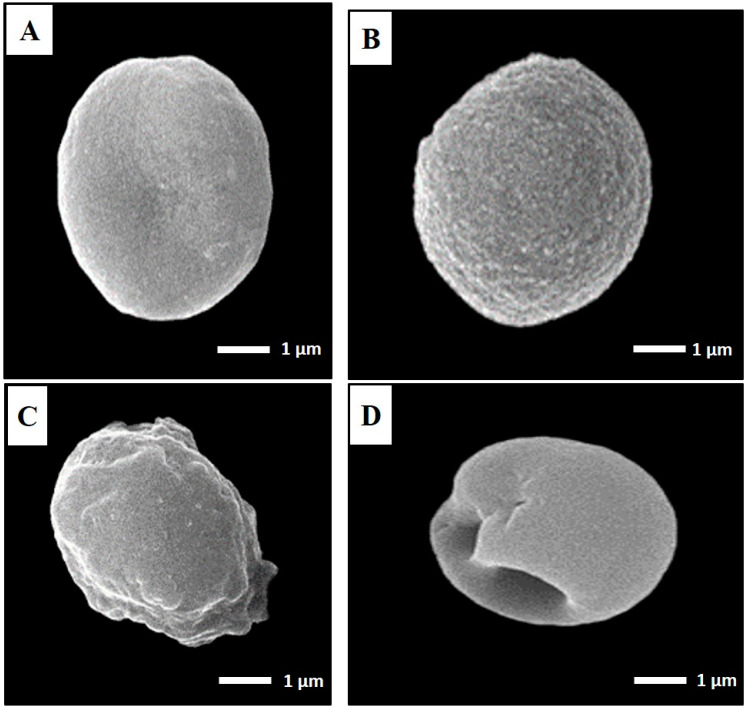
SEM ultrastructural morphological features of *T. gondii* cysts isolated from the infected control group (**A**), mice preimmunized with TLA (**B**), and mice preimmunized with TLA/RA-SLNs (**C**,**D**) (×10,000). (**A**) Regular spherical-shaped bodies. (**B**) Mild-to-moderate roughness and surface irregularities yet preserved spherical outline. (**C**) Marked surface irregularities with diffuse swelling. (**D**) Body indentations.

*Toxoplasma* cyst SEM:

Using SEM, *T. gondii* cysts in the brain homogenate from infected, nonvaccinated mice and those vaccinated with either TLA alone or TLA/RA-SLNs were examined four weeks following challenging the infection (Figure 5). *T. gondii* cysts isolated from the control group were spherical in shape, with different sizes. Their surface looked regular, with some fine indentations (Figure 5A). Cysts isolated from preimmunized mice with the aforementioned protocols, on the other hand, showed varying grades of morphological alterations. *T. gondii* cysts isolated from mice preimmunized with TLA alone had rough surfaces with fine granulations yet maintained their spherical shape (Figure 5B), while those harvested from mice preimmunized with TLA combined with RA-SLNs exhibited marked surface irregularities, diffuse swelling, and body indentations (Figure 5C,D).

#### 3.2.2. Immunological Results

**Figure 6:**
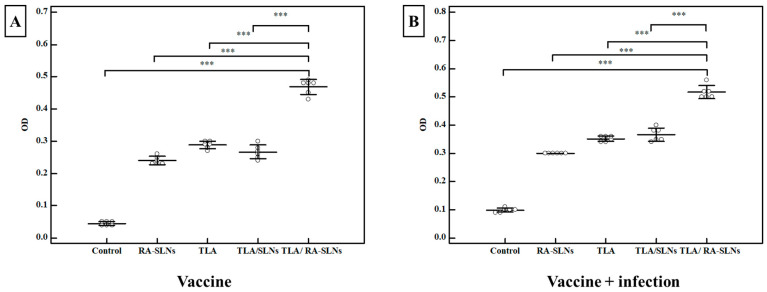
The *Toxoplasma*-specific IgG titers in sera (OD) among various evaluated regimens. (**A**) The total IgG antibody titers in mice sera two weeks after the second booster dose of immunization (vaccine only). (**B**) The total IgG antibody titers in mice sera at the 4th week following infection challenge (vaccine + infection). Data were conveyed by using mean ± SD. ***: statistically significant at *p* ≤ 0.001.

Total IgG level in serum:

Concerning the IgG titers following complete immunization protocols and before the infection, all evaluated schedules induced a significant increase in IgG titer when compared to the control group, which had a mean IgG level of 0.034 ± 0.007 OD (*p* < 0.001) (Figure 6). TLA combined with plain SLNs resulted in a nonsignificant increase in IgG titers when compared to the group that received TLA alone. In contrast, mice inoculated IN with TLA combined with RA-SLNs resulted in the greatest increase in IgG among different evaluated regimens, with a mean of 0.469 ± 0.023 OD. Similarly, inducing infection using the oral challenge of mice with avirulent ME49 *Toxoplasma* cysts encouraged the production of anti-*Toxoplasma* IgG, which retained its high titers across different evaluated regimens in the same manner recorded during the pre-infection period (Figure 6).

**Figure 7:**
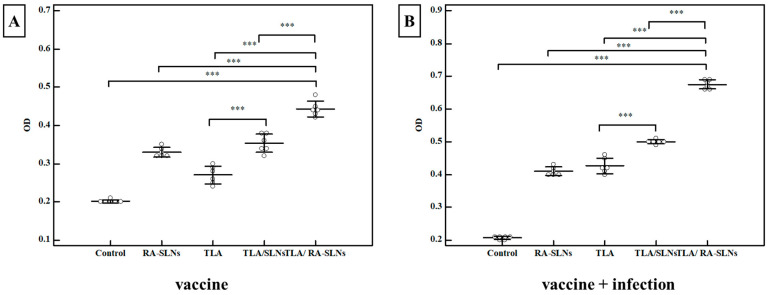
The *Toxoplasma*-specific IgA titers (OD) in the intestinal washes of various assessed protocols. (**A**) *Toxoplasma* S-IgA antibody titers in mice two weeks following immunization schedule’s completion (vaccine only). (**B**) *Toxoplasma* S-IgA antibody titers in mice at week 4 after infection (vaccine + infection). Data were conveyed by using mean ± SD. ***: statistically significant at *p* ≤ 0.001.

Secretory IgA level in the intestinal wash:

Regarding the IgA levels before infection, the control group showed a mean anti-*Toxoplasma* IgA level of 0.201 ± 0.004 OD. In comparison to the normal control group, all of the evaluated regimens revealed a significant rise in the induction of IgA at various levels (Figure 7). An enhancement in mucosal immune response was recorded in mice preimmunized with TLA/plain SLNs; however, adjuvanting TLA with RA-SLNs resulted in the greatest significant IgA titers, with a mean of 0.442 ± 0.021 OD. Following the infection, the anti-*Toxoplasma* IgA levels significantly increased in all evaluated regimens compared to the infected control group. However, mice preimmunized with TLA/RA-SLNs demonstrated the greatest increase in anti-*Toxoplasma* IgA level among various evaluated regimens with a mean level of 0.675 ± 0.015 OD (Figure 7).

**Figure 8:**
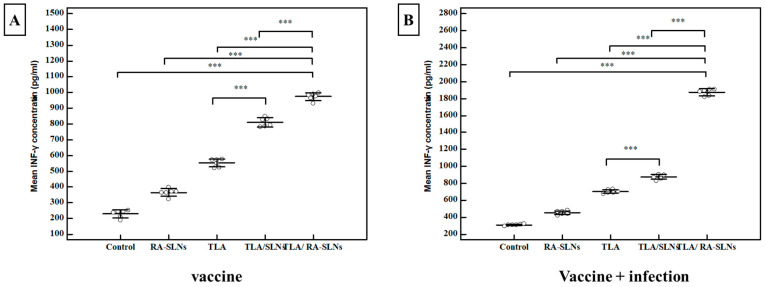
The IFN-γ concentrations among various assessed protocols. (**A**) INF-γ concentration (pg/mL) two weeks after the final immunization dose (vaccine). (**B**) IFN-γ concentration (pg/mL) at the 4th week after infection (vaccine + infection). Data were expressed by using mean ± SD. ***: statistically significant at *p* ≤ 0.001.

Interferon gamma (IFN-γ) level:

Splenic cell suspensions from six mice in each group were cultured and stimulated in vitro with TLA; after that, IFN-γ levels in the culture supernatants were measured using ELISA. Regarding the IFN-γ levels before infection (vaccine only), the mean concentration of IFN-γ was 231 ± 24.5 pg/mL in the control group (I). In comparison to the normal control group, all studied regimens significantly potentiated IFN-**γ** production at various levels (Figure 8). The RA-SLNs increased the efficacy of the TLA with a mean IFN-γ concentration of 973 ± 24.3 pg/mL. As a result, the mean IFN-γ concentration increased significantly when compared to the control groups. Similarly, after challenging infection, all studied regimens demonstrated an enhancement in mean IFN-γ concentration when compared to the infected control group in the same manner as previously described in the pre-infection period (Figure 8). 

#### 3.2.3. Biochemical Results

The control group brain SOD levels showed a mean of 1415.33 ± 9.7 U/g tissue two weeks after the last booster dose with no statistically significant differences in the mean SOD levels among various evaluated regimens compared to the normal control group at this point in the immunization schedule. In contrast to the aforementioned findings, challenging infection with the tissue cyst of *T. gondii* ME49 resulted in a significant increase in SOD level in all evaluated regimens. The infected control group had an increase in SOD mean level, reaching 1541 ± 16.4 U/g tissue. Comparable to this measure, a statistically significant increase in mean SOD levels was recorded with superiority of the TLA/RA-SLN group in all evaluated regimens. It revealed a mean SOD level of 2018 ± 18.7 U/g tissue (Table 2).

Regarding brain MDA levels, the control group had a mean pre-infection MDA level of 16.92 ± 0.70 nmol/mg. Mice preimmunized with TLA/RA-SLNs exhibited the lowest MDA level among different studied groups, as this regimen induced a mean MDA level of 15.08 ± 0.21 nmol/mg. On the other hand, none of the other evaluated protocols produced any significant changes when compared to the control group (Table 2). The mean MDA level in the infected control group brain homogenates increased after infection, reaching 29.70 ± 0.59 nmol/mg. Similarly, mice preimmunized with TLA alone or in combination with plain SLNs also had higher mean MDA levels when compared to pre-infection values. On the other hand, the use of RA-SLNs alone or in combination with TLA prevented the increasement in the MDA levels, with a mean of 11.03 ± 0.34 and 13.85 ± 0.39 nmol/mg, respectively (Table 2).

Regarding serum TAC levels, the control group had a pre-infection mean TAC level of 0.99 ± 0.03 mM/L. As shown in Table 2 the sera levels of TAC in all evaluated regimens increased significantly when compared to the control group. As a consequence of combining RA-SLNs to TLA, the TAC level increased significantly up to 1.47 ± 0.02 mM/L. Following the oral inoculation of mice with the avirulent *T. gondii* ME49 tissue cysts, mice from all evaluated regimens maintained the same statistically significant elevation of serum TAC levels when compared to the infected control group or to each other (Table 2).

#### 3.2.4. Histopathological Results

**Figure 9:**
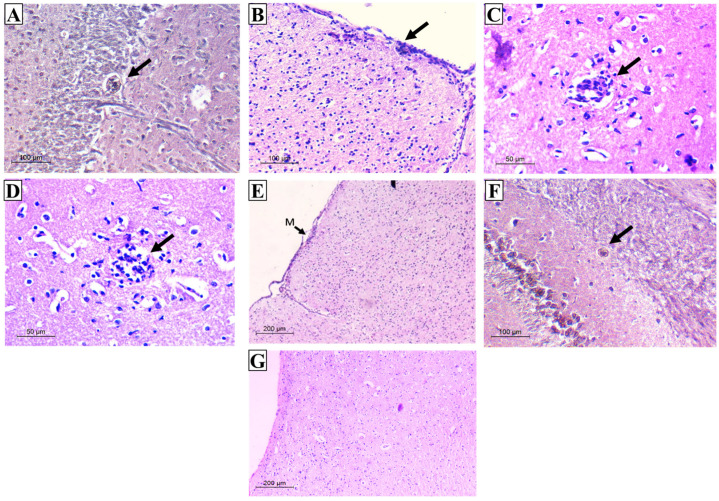
Histopathological outcomes in the examined brain sections of the infected control group (**A**–**E**) and vaccinated groups (**F**,**G**). (**A**) GMS section displaying rounded *T. gondii* cyst with well-defined intact wall in the cerebellum (×200); (**B**) H&E section displaying severe meningitis on cerebral cortex surface (×200); (**C**) H&E section displaying perivascular infiltration (×400); (**D**) H&E section displaying aggregates of microglial cells (×400); (**E**) H&E section displaying hypercellularity all over the brain tissue (M: meninges) (×100); (**F**) GMS section displaying very small cyst with thin wall and few bradyzoites (×200); (**G**) H&E section displaying the upgrading of inflammatory scores with the absence of either meningeal infiltration or cerebral-increased cellularity (×100).

Using Grocott–Gomori methenamine silver stain (GMS), a well-defined intact cyst wall surrounding the *Toxoplasma* cyst was obviously seen (Figure 9A). Most of these cysts were located in the cerebellum and cerebral cortex in areas of normally appearing neural tissues that were not associated with inflammatory nodules. Nevertheless, H&E-stained sections revealed a variety of histological lesions, including frequent perivascular and leptomeningeal infiltrations with inflammatory cells commonly found on the cerebral cortex’s surfaces (Figure 9B,C). These inflammatory cellular infiltrates primarily comprised lymphocytes and few histiocytes. Furthermore, throughout the cerebral parenchyma, large aggregates of microglial cells and astrocytes with hypercellularity were evident (Figure 9D,E). On the other hand, mice preimmunized with various evaluated regimens showed various levels of upgraded inflammatory scores where residual focal aggregates of microglial cells and trivial meningeal affection were recorded. Furthermore, cysts appeared very small with thin cyst walls and few bradyzoites (Figure 9F). Interestingly, mice preimmunized with TLA/RA-SLNs (group V) revealed a significant improvement in histopathological outcomes. No evidence of perivascular or leptomeningeal affections was detected. Aggregates of microglial cells were scarcely found. (Figure 9G). 

## 4. Discussion

Vaccines are among some of the most impactful public health advances to date, playing a crucial role in minimizing the spread of the world’s many devastating infectious diseases [36]. However, the majority of the new potential vaccines have low immunogenicity, meaning they are unable to elicit powerful and long-lasting immune responses [37]. Therefore, adjuvants and innovative delivery systems are thus critical for the development of modern and efficacious vaccines [37]. Various reports have revealed the part played by antioxidants in ameliorating the adverse effects of vaccines, in addition to their significant role in disease management, especially those mediated by ROS such as toxoplasmosis [38]. Among these, RA has gained particular interest as a natural element with known antioxidant effects as well as immunomodulatory effects [39,40]. Thus, this study experimentally investigated the prospective role of nano-encapsulated RA as a novel adjuvant to boost the anti-*Toxoplasma* vaccine’s protective immunity, hand in hand with their ability to modulate the pathology imposed by parasitic infection as a natural antioxidant and anti-inflammatory agent.

Physicochemical analysis of the manufactured nanodispersions unveiled that the encapsulation of RA in SLNs caused an increase in particle diameters, which could be explained by the preparation’s high lipid/adjuvant ratio (10:1) required to improve RA incorporation within SLNs. Much of the literature has described that the diameter of lipid nanoparticles is greatly reliant on the used amount of lipid that can be clarified in terms of the affinity of the lipid to amalgamate at increased lipid concentration forming larger sized particles [41,42]. On the other hand, ZP specifies the degree of electrostatic repulsion between nearby and similarly charged particles in a dispersion, which is a crucial factor for evaluation of the stability of a colloidal dispersion. The incorporation of stearylamine in the lipid composition of the prepared nanoparticles increased ZP values, rendering a positive outcome of both RA-SLNs and its plain form [23]. The achieved high ZP values for the prepared SLN formulations give rise to their great physical stability and their ability to overcome agglomeration upon storage. Similar results were reported in previous studies [23,27]. The aforementioned results of the colloidal characterization of the manufactured nanodispersions were supported by the electron-microscopic study of the freshly prepared particles where they were completely spherical in shape with smooth surfaces and well dispersed with good particle size distributions.

The high EE of RA in SLNs was owed to the ion pairing formation between RA and the lipophilic amine (stearylamine) that enhanced the lipophilic nature of RA, rendering it easily encapsulated into the lipid matrix. This was carried out to overcome the low encapsulation of RA in the SLNs that requires an increased surfactant/lipid ratio to be used, which advocates the adjuvant position at the water–oil interface, owing to the RA’s high amphiphilicity, and diminishes the benefits gained by its incorporation into the lipid matrix such as improved stability, controlled release, and targeting effect [22,23]. Furthermore, the addition of cosurfactant as cholesterol also improved EE by providing a greater superficial area and favored the interfacial adsorption of RA [23]. Moreover, the introduction of cholesterol to the lipid matrix structure increased the number of lattice defects, which is related to easier adjuvant incorporation in SLNs [23]. Lattice defects are part of a crystal lattice where the repeating structure is disrupted, creating missing atoms or lattice misalignment. In the nanoparticle structure, adjuvant expulsion occurs from the nanoparticle structure of the lipid results in a highly crystalline structure with a perfect lattice. On the other hand, the lipid structure’s imperfections (lattice defects) may provide extra loading space to accommodate the adjuvant [43].

The in vitro release of RA-SLNs at 37 °C revealed controlled, sustained adjuvant release comparable to its free form. This could be attributed to the high surfactant content that stabilizes the SLNs; it also limits the amount of adjuvant released outside the SLNs, permitting the sustained release [44]. Additionally, increasing the lipid amount increased the viscosity of the medium, resulting in more firm, hardened nanoparticles that delayed the adjuvant dispersion to the external medium [42]. As a result, RA encapsulation is superior to its free form regarding its enhanced bioavailability and improved in vivo half-life.

Additionally, RA-SLNs revealed a desirable stability that was gained by employing RA-STE ion pairing [23]. Our results indicated that the dispersions had a long shelf life, implying that RA-SLN formulation could be an encouraging adjuvant delivery system for a *Toxoplasma* vaccine.

In terms of adjuvant regimen safety, it has been reported that adjuvants can provoke a tailor-made, dose-dependent immune response to a particular pathogen [45]. RA was administered at a very low concentration of 15 µg/dose. This dose was reported to be safe and potent in intranasal immunization against cutaneous leishmaniasis [27].

In the present experiment, the intranasally administered RA-SLNs augmented the TLA immunogenicity in the defense against toxoplasmosis, as proven by the studied parasitological, immunological, antioxidant, and histopathological parameters. This enrichment could be elucidated by the fact that TLA alone does not provide protection against toxoplasmosis [46]. This may be attributed to the fact that, without concurrent administration of effective adjuvants, most killed or inactivated vaccines are weak enhancers of adaptive immunity [47].

Several pieces of evidence support the association of antibodies in the defense against pathogens using diverse methods, such as inhibiting the attachment to host cells [48]. Much of the literatures has shown that *Toxoplasma*-specific antibodies can block the in vitro penetration of enterocytes by tachyzoites [49,50,51]. Thereby, in the current study, the capability of the harvested sera from mice two weeks following the completion of different immunization regimens to prevent in vitro parasite invasion was assessed. Our results showed that sera derived from mice preimmunized with TLA, either alone or in combination with RA, reduced tachyzoite invasion and replication in Vero cells in vitro to varying degrees. These findings are consistent with those reported before that sera obtained from TLA-vaccinated mice reduced tachyzoite invasion and replication [7]. Interestingly, mice preimmunized with TLA/RA-SLNs recorded the uppermost reduction, indicating that this regimen elicited the strongest antibody response.

Mice preimmunized with various schedules resulted in a significant decrease in brain cyst load in all evaluated regimens compared to the control. Moreover, it was obvious that RA, rather than plain SLNs, is the principal booster of TLA immunogenicity. It was noticed that mice preimmunized with TLA/RA-SLNs had a significantly lower brain cyst count and cyst size when compared to the TLA group, while no significant alteration was found in mice preimmunized with TLA either alone or combined with plain SLNs. Humoral, mucosal, and cellular immune responses in various studied regimens were analyzed to corroborate the probable explanations of this dominance. 

The current results revealed that TLA in combination with plain SLNs caused a non-significant change in IgG levels when compared to its sole administration (TLA alone). In contrast, Shirai et al., 2020, reported that lipid nanoparticles had an adjuvant role and could improve IgG responses with superior protection against the influenza virus challenge [28]. The reason for the conflicting findings could be related to the different SLNs’ formulation and size used in the present experiment from that used in the previous one, as well as the diverse types of antigens used. 

When administered alone or combined with TLA, RA-SLNs significantly increase serum anti-*Toxoplasma* IgG levels either before or after infection. This demonstrated that, when associated with the antigen of interest, RA functioned as an effective adjuvant to B cell epitopes.

RA enhanced the humoral response, which is in corroboration with other previous studies [52,53]. RA is crucial for B cell production, maturation, and differentiation into plasma cells. It has been reported to be an important cofactor for the stimulation and proliferation of B cells by hastening B cell lymphopoiesis through increasing the peripheral B cell count in the spleen while reducing lymphoid precursors in the marrow [54]. Therefore, RA can induce an efficient, enduring humoral response through the growth of germinal centers (GCs) in the lymphoid follicles, allowing interactions between B cells and follicular helper T cells that ensure the development of memory B cells and enduring plasma cells [55]. Therefore, B cells go through somatic hypermutation and immunoglobulin class switching recombination as a part of the GC reactions [55]. Additionally, it has a role in induction of interferon regulatory factor 4 (IRF4) expression, which is tangled in the development of plasma cells and RA-mediated IgG generation [56].

It is now widely reported that anti-*Toxoplasma* IgG antibodies could stop infection by inhibiting parasite duplication and adhesion to host cell receptors, while facilitating adhesion to immune cells and promoting intracellular death by macrophages through antibody-dependent cell-mediated cytotoxicity (ADCC) [57]. 

Considering that the intestinal mucosa is the gateway for *T. gondii* access, stimulation of an effective local response at this level represents an utmost that could be obtained by the utilization of nasal vaccines. In the current work, enhancement of mucosal immune response was noted by combining TLA with plain SLNs or RA-SLNs, which recorded the highest significant increase in IgA titers at various time intervals (before and after infection).

Mucosal IgA is essential for maintaining mucosal homeostasis that could aid in pathogen eradication and limit the spread of *T. gondii* to more tissues, particularly the muscles and brain [58]. Because of its ability to transcytose across epithelial cells, pathogen-specific IgA is the most abundantly existing antibody isotype that plays a key role as the first line of defense against infectious diseases on the mucosal surfaces [59,60]. Additionally, mucosal IgA comprises antibodies that identify antigens with high and low affinity-binding manners; it is thereby able to counteract inflammatory microbial products inside epithelial cells combined with the capability to and agglutinate microorganisms to facilitate their clearance by mucociliary flow or peristalsis [61,62]. Furthermore, secretory IgA plays a role in protection against *Toxoplasma* after natural infections by hindering tachyzoite infection of host cells and impeding *T. gondii* replication in enterocytes during transcytosis. Therefore, the generation of efficacious mucosal immune responses at gut level against toxoplasmosis could prevent the severity of such infection [63]. 

Our results are in agreement with those pointing to the capability of the high pre-infection levels of intestinal IgA, in preimmunized mice, to counteract or block the orally administrated *Toxoplasma* cysts [8]. The documented role of RA in promoting the immunoglobulin switch to IgA isotype was evidenced in the present study, where mice vaccinated with TLA/RA-SLNs (group III) demonstrated the highest significant upsurge in IgA titers before infection (vaccine only). We assumed that the combined action of the elevated pre-infection IgA and post-infection IgG might contribute to the highest statistically significant reduction in cyst count and size encountered in this group.

Cytokines are an alternative important line of parasite defense, apart from antibodies, that are critical in synchronizing both innate and adaptive host defense mechanisms during *T. gondii* infection. In the present study, IFN-γ was used as an indicator for cellular immunity [7]. RA-SLNs augmented the efficacy of TLA with the highest statistically significant elevation of IFN-γ concentration in culture supernatant of splenocyte suspension among the studied groups. This could be attributed to the amplified, specific, and robust responses generated by RA-SLNs in combination with a particular vaccine antigen (TLA) owing to their capacity to precisely target the dendritic cells (DCs), resulting in an enhancement of cellular uptake and antigen presentation [64]. Abhyankar et al., 2017, demonstrated that the inclusion of RA in vaccination against *Entamoeba histolytica* elicited a robust increase in IFN-γ [65]. Similar findings were reported by Riccomi et al., 2019, who revealed that vaccination with a tuberculosis (TB) subunit vaccine in presence of RA elicited higher amounts of IFN-γ cytokines in lung homogenates of vaccinated mice [66].

It is well known that the ability of any vaccine to enhance powerful and comprehensive systemic Th1 cell response affords defensive immunity against *T. gondii* infection. Moreover, Th1 cellular immunity is primarily dependent on T cells’ ability to produce IFN-γ, which controls both acute and chronic *Toxoplasma* infection. IFN-γ restricts parasite growth in the acute phase, in addition to its role in preventing the reactivation of bradyzoites in the chronic phase via IFN-γ-mediated NO release in various host cells including macrophages [67]. In addition, host resistance to *T. gondii* infection can be mediated by IFN-γ via induction of STAT-1-dependent expression of several antimicrobial genes and p47 GTPases. The latter is a particular class of proteins that has been widely involved in the induction of protection from intracellular pathogens in mice and mediating the cell-autonomous fight against *T. gondii* [68]. Moreover, p47 GTPases accumulate around vacuoles containing *T. gondii* in activated astrocytes and initiate the disruption of the parasitophorous vacuole and eventually the destruction of the parasite itself [69]. IFN-γ is mainly produced by CD8+ cytotoxic T cell populations, which are considered the primary effector cells against *T. gondii* [70]. It was recently reported that CD8+ T cells are able to remove *T. gondii* tissue cysts through perforin from their lytic granules and implanted into the cyst wall to form pores that expedite cyst invasion and subsequent eradication. The pore-forming action of perforin causes harm to the cyst wall; thereby, cysts invaded by T cells exhibited morphologic deterioration and destruction [71].

The previously mentioned immunological results were supported by the ultrastructural changes observed in a considerable number of *T. gondii* cysts harvested from mice preimmunized with either TLA alone or combined with RA-SLNs. These deformities might be ascribed to the stout cellular immunity. It was reported that CD8+ cytotoxic T cells are able to penetrate the *Toxoplasma* cysts, as they were visualized across the cyst wall and even fully situated within the cysts in the brains of infected mice. They exert their direct cyst removal effect through perforin-mediated cyst burden reduction together with morphological changes [71]. Tiwari et al., 2019, assumed that the observed inward bending of the cyst wall is mostly caused by the pressure exerted by the CD8 cytotoxic T cells in order to penetrate into the cysts [71].

The host immune response against infection results in intensive production of ROS with induction of OS. In spite of the important role of ROS in the fight against *T. gondii* infection, the buildup of potentially harmful levels of ROS resulted in a deleterious effect, imposing tissue insults [72]. Moreover, the antioxidant/oxidant balance has a strong influence on immune cell functions. Additionally, the brain is good prey for unwarranted oxidative insults since it has elevated energy requirements, copious lipid content, and weak antioxidant capacity with augmented vulnerability to neuronal injury and functional deterioration via brain oxidation [73]. Therefore, the inclusion of antioxidants in vaccine formulation against *T. gondii* is critical for either defense against oxidative damage or conservation of adequate cellular function [74]. Moreover, producing an antioxidant response capable of retaining oxidant agents at low concentrations to avoid IgG oxidation would greatly benefit any vaccine formulation. Contreras et al., 2020, perceived that low oxidant biomarkers were associated with a rise in specific IgG antibodies, which possibly leads to less impairment of immunoglobulins or even a more effective generation of those IgGs [75].

One of the most important cornerstones of the antioxidant defense system is SOD, which is considered the primary line of defense against ROS. Preimmunization of mice with TLA combined with RA-SLNs produced the greatest significant levels of SOD, revealing the key role of this adjuvant as a prospective modulator of antioxidant defense together with its direct immunomodulatory effect. Researchers have previously reported that RA produced a substantial rise in neuronal survival, reduced neuronal ROS content, and enhanced protein levels of SOD-1 and SOD-2 supporting the antioxidant defense system and reducing staurosporine-induced oxidative stress [76]. Moreover, Elomda et al., 2018, highlighted the potential impacts of RA as an antioxidant in the culture medium on the development of rabbit embryos in vitro with significantly higher SOD activity and upregulation of the gene expression of SOD-1 [77].

The brain is easily vulnerable to damage from elevated OS by lipid peroxidation, where a single priming free radical can trigger the damage of nearby molecules, owing to the existence of an elevated concentration of polyunsaturated fatty acids in its membrane lipids [78]. Moreover, membrane receptors, which are required for antigen recognition, can also be damaged by membrane lipid peroxidation [79]. At pre-infection levels, none of the studied regimens exhibited a statistically significant difference in MDA levels except in TLA/RA-SLNs group (group V). Kayalar et al., 2013, demonstrated that RA offers a better environment for the brain against OS [80]. They reported that despite the increased endogenous antioxidant mechanisms of the adult mice brain in response to OS, this was not enough to reduce lipid peroxidation and ROS elimination. However, RA hindered excessive lipid peroxidation in the brain [80].

Following infection, brain homogenates of infected control group recorded elevated MDA levels. This was as anticipated, because much of the literature has declared that the elevated amounts of MDA in chronic toxoplasmosis can reflect the level of oxidative cell damage indirectly [81]. Likewise, vaccination of mice with TLA alone or adjuvanted with plain SLNs also showed an elevation in the mean MDA level when compared to the pre-infection level. This indicated their inability to counteract the infection-induced increase in lipid peroxidation. It was observed that ROS levels increased in TLA-vaccinated mice, signifying its oxidant potential in a study performed by Fatollahzadeh et al., 2021 [82].

The mean MDA levels in brain homogenates of mice pre-immunized with RA-SLNs alone or combined with TLA were significantly reduced. However, better reduction was observed in regimens including RA encapsulated in SLNs alone. This could be attributed to the oxidant effect of TLA in group V that may partially decrease the antioxidant effect of RA, which is a small lipophilic molecule that was reported to reduce OS in the brain by easily crossing the BBB, resulting in a significant reduction in lipid peroxidation in hyperoxic mice [83]. Additionally, RA prevents the upsurge in MDA through the removal of reactive hydroxyl and peroxyl radicals, as reported by Jiang et al., 2007 [84].

Similarly, the TLA/RA-SLNs group had the greatest significant rise in TAC level, as expected, given its successful influence on the antioxidants and enzymatic activity, as well as its documented capacity to enhance SOD gene expression levels leading to less ROS accumulation and a higher level of TAC [77].

The objective of any immunization regimen should entail the modulation of the pathology induced by the infectious agent rather than focusing only on protection against infection. Therefore, the histopathological consequences of the chosen antioxidant, immunomodulatory and anti-inflammatory adjuvant, and its role in limiting brain pathology were assessed. In the current study, amelioration of infection-induced inflammatory changes in the brain was achieved in different studied regimens when compared to the control group. Vaccination of mice with TLA combined with RA-SLNs revealed mild inflammatory changes where perivascular inflammation or glial nodules were hardly detected. This could be attributed to the direct immunomodulatory and antioxidant effects of RA and its indirect effect through the reduction in brain cysts.

The ability of the chosen adjuvant to reduce inflammatory changes, especially overactivation of microglia, reflected its direct immunomodulatory effect, which could be very promising in future vaccine development. In this work, unlike the brains of vaccinated mice, the brains harvested from control infected mice showed severe inflammatory changes together with many aggregates of microglial cells and astrocytes. Although activated microglia were reported to have a role in fighting infection, as they are considered the primary line of defense against CNS-invading pathogens, thus contributing to both forms of immune response, their continued existence throughout the chronic state is detrimental to the brain [85]. In fact, most CNS insults are frequently connected with aberrant microglial stimulation [85]. 

Additionally, the improvement in OS gained by the used antioxidant adjuvant protected the cells from free radical damage with obvious upgrading in brain inflammatory scores. This is in agreement with emerging research evidence, which has proposed that infection-induced OS is the main mechanism involved in the development of TE and apoptosis through pathological levels of ROS generated from activated microglia/macrophages [14]. Both internal and external apoptotic pathways have been reported to be activated by oxidative stress [14]. This was supported by previous finding declaring that SOD upregulation in neurons causes an interruption in apoptosis [86]. The current study found that the nano-encapsulated antioxidant adjuvant had neuroprotective and anti-inflammatory effects, which could be explained by its delivery route (IN), which in turn promoted its brain delivery directly from the nose along the olfactory and trigeminal nerves.

## 5. Conclusions

This experimental work is intended to shine a spotlight on the prospective role of RA as an immunomodulator and a natural antioxidant in the fight against one of the most prevalent infectious diseases, toxoplasmosis, which could invigorate adjuvant development and design due to its multitarget capacities. The present work provides an appealing experimental endeavor to promote and improve the efficacy of TLA with this adjuvant while reducing the proportion of undesirable events of already existing ones such as alum, which could be attributed to the chemical composition and interactions. The findings presented here demonstrate the critical antioxidant role of such a compound hand in hand with its immunomodulatory mechanisms to compete with the infection-induced OS, which ensures vaccine safety and efficacy by attenuating the detrimental impacts of *T. gondii* infection. Furthermore, together with the success of the intranasal route in providing protection against chronic toxoplasmosis, SLNs proved to be a reliable adjuvant delivery platform. These findings could open up new avenues for the use of RA in forthcoming immunotherapies and immunoprophylaxis strategies.

## Figures and Tables

**Table 1:** Physicochemical properties of RA-SLN suspensions after storage at 4 °C for the studied durations.

RA-SLNs at 4 °C	Zero (n = 3)	1 Month (n = 3)	2 Months (n = 3)	3 Months (n = 3)	6 Months (n = 3)	F	*p*
**Size (nm)**	326 ± 6	327 ± 3	328 ± 4	328 ± 5	330 ± 2	0.367	0.827
**PDI**	0.27 ± 0.3	0.27 ± 0.1	0.27 ± 0.03	0.27 ± 0.1	0.27 ± 0.07	0.003	1.000
**ZP (mV)**	47.0 ± 0.6	46.0 ± 0.5	47.0 ± 0.5	47.0 ± 0.7	46.0 ± 0.6	2.632	0.098 *
**EE (%)**	89.1 ± 0.6	89 ± 0.03	89.3 ± 0.1	88.9 ± 0.1	88.8 ± 0.7	0.637	0.648

Data were expressed by using mean ± SD. Zero day: freshly prepared suspension; n: number of samples; PDI: polydispersity index; EE: encapsulation efficiency; F: F for one-way ANOVA test; *p*: *p*-value for comparing between the different studied times; *: statistically significant at *p* ≤ 0.05.

**Table 2:** The mean SOD (U/g tissue), MDA (nmol/mg) levels in brain homogenates, and TAC levels (mM/L) in sera of various evaluated regimens at different periods.

	Group I (n = 6)	Group II (n = 6)	Group III (n = 6)	Group IV (n = 6)	Group V (n = 6)	^F^ *p*
**SOD**						
Pre-infection	1415 ± 9.7	1424 ± 5.8	1426 ^a^ ± 4.5	1428 ^a^ ± 3.4	1430 ^a^ ± 6.1	0.006 *
Post-infection	1541 ± 16.4	1739 ^a^ ± 26.2	1753 ^a^ ± 9.4	1658 ^abc^ ± 23.2	2018 ^abcd^ ± 18.7	<0.001 *
**MDA**						
Pre-infection	16.92 ± 0.70	17.33 ± 0.64	16.98 ± 0.55	17.13 ± 0.35	15.08 ^abcd^ ± 0.21	<0.001 *
Post-infection	29.70 ± 0.59	11.03 ^a^ ± 0.34	22.88 ^ab^ ± 0.81	22.67 ^ab^ ± 0.30	13.85 ^abcd^ ± 0.39	<0.001 *
**TAC**						
Pre-infection	0.99 ± 0.03	1.32 ^a^ ± 0.04	1.45 ^ab^ ± 0.03	1.42 ^ab^ ± 0.02	1.47 ^abd^ ± 0.02	<0.001 *
Post-infection	1.26 ± 0.02	1.45 ^a^ ± 0.03	1.52 ^ab^ ± 0.02	1.53 ^ab^ ± 0.02	1.59 ^abcd^ ± 0.01	<0.001 *

SD: standard deviation; ^F^: F for ANOVA test. Pairwise comparison between every two groups was conducted using post hoc test (Tukey). *p*: *p*-value for comparing between the different studied groups. a: statistically significant with group I. b: statistically significant with group II. c: statistically significant with group III. d: statistically significant with group IV. *: statistically significant at *p* ≤ 0.05.

## Data Availability

Not applicable.

## Supplementary Materials

The following supporting information can be downloaded at: https://www.mdpi.com/article/10.3390/tropicalmed8020106/s1. Table S1: Mean number of *T. gondii* tachyzoites (×10^4^) per well in culture supernatant of Vero cells among all studied groups.
