# Supplementary material for: Nano-Encapsulated Antioxidant: Retinoic Acid as a Natural Mucosal Adjuvant for Intranasal Immunization against Chronic Experimental Toxoplasmosis
Source: Trop Med Infect Dis. 2023 Feb 7;8(2):106. doi: 10.3390/tropicalmed8020106 (PMC9962073; doi:10.3390/tropicalmed8020106)
Supplement: Supplementary file 1 [file tropicalmed-08-00106-s001.zip › tropicalmed-2103702-supplementary.pdf]

Table S1: Mean number of *T. gondii* tachyzoites (× 10^4) per well in culture supernatant of Vero cells among all studied groups:

Tachyzoite count	Group I (n = 3)	Group II (n = 3)	Group III (n = 3)	Group IV (n = 3)	Group V (n = 3)	^{F}p
Mean	46.67	33.33	25.17	25.67	16.33	$<0.001^*$
±SD.	3.06	1.15	1.26	0.58	1.53	
**% reduction**		28.6	46.1	45.0	65.0	
p_1		$<0.001^*$	$<0.001^*$	$<0.001^*$	$<0.001^*$	
p_2			0.001^*	0.002^*	$<0.001^*$	
**Sig. bet. grps.**	$p_3 = 0.996$, $p_4 = 0.001^*$, $p_5 < 0.001^*$					

SD: **Standard deviation**, F: **F for ANOVA test**, Pairwise comparison bet each 2 groups was done using **Post Hoc Test (Tukey)**

p: p value for comparing between the different studied groups

p_1; p value for comparing between **Group I** and each other group

p_2; p value for comparing between **Group II** and each other group

p_3; p value for comparing between **Group III** and **Group IV**

p_4; p value for comparing between **Group III** and **Group V**

p_5; p value for comparing between **Group IV** and **Group V**

*: Statistically significant at $p \leq 0.05$

Group I: Control **Group II**: RA–SLNs, **Group III**: TLA

Group IV: TLA/ plain SLNs, **Group V**: TLA/ RA–SLNs,
